# Cheese Ingestion Increases Muscle Protein Synthesis Rates Both at Rest and During Recovery from Exercise in Healthy, Young Males: A Randomized Parallel-Group Trial

**DOI:** 10.1093/jn/nxac007

**Published:** 2022-01-10

**Authors:** Wesley J H Hermans, Cas J Fuchs, Floris K Hendriks, Lisanne H P Houben, Joan M Senden, Lex B Verdijk, Luc J C van Loon

**Affiliations:** Department of Human Biology, NUTRIM School of Nutrition and Translational Research in Metabolism, Maastricht University, Maastricht, The Netherlands; Department of Human Biology, NUTRIM School of Nutrition and Translational Research in Metabolism, Maastricht University, Maastricht, The Netherlands; Department of Human Biology, NUTRIM School of Nutrition and Translational Research in Metabolism, Maastricht University, Maastricht, The Netherlands; Department of Human Biology, NUTRIM School of Nutrition and Translational Research in Metabolism, Maastricht University, Maastricht, The Netherlands; Department of Human Biology, NUTRIM School of Nutrition and Translational Research in Metabolism, Maastricht University, Maastricht, The Netherlands; Department of Human Biology, NUTRIM School of Nutrition and Translational Research in Metabolism, Maastricht University, Maastricht, The Netherlands; Department of Human Biology, NUTRIM School of Nutrition and Translational Research in Metabolism, Maastricht University, Maastricht, The Netherlands

**Keywords:** stable isotope tracers, protein metabolism, muscle metabolism, whole foods, dietary protein, dairy, fermented dairy, food processing, food matrix, healthy young males

## Abstract

**Background:**

Protein ingestion increases muscle protein synthesis rates. The food matrix in which protein is provided can strongly modulate the postprandial muscle protein synthetic response. So far, the muscle protein synthetic response to the ingestion of whole foods remains largely unexplored.

**Objectives:**

To compare the impact of ingesting 30 g protein provided as milk protein or cheese on postprandial plasma amino acid concentrations and muscle protein synthesis rates at rest and during recovery from exercise in vivo in young males.

**Methods:**

In this randomized, parallel-group intervention trial, 20 healthy males aged 18–35 y ingested 30 g protein provided as cheese or milk protein concentrate following a single-legged resistance-type exercise session consisting of 12 sets of leg press and leg extension exercises. Primed, continuous intravenous L-[*ring*-^13^C_6_]-phenylalanine infusions were combined with the collection of blood and muscle tissue samples to assess postabsorptive and 4-h postprandial muscle protein synthesis rates at rest and during recovery from exercise. Data were analyzed using repeated measures Time × Group (× Leg) ANOVA.

**Results:**

Plasma total amino acid concentrations increased after protein ingestion (Time: *P* < 0.001), with 38% higher peak concentrations following milk protein than cheese ingestion (Time × Group: *P* < 0.001). Muscle protein synthesis rates increased following both cheese and milk protein ingestion from 0.037 ± 0.014 to 0.055 ± 0.018%·h^–1^ and 0.034 ± 0.008 to 0.056 ± 0.010%·h^–1^ at rest and even more following exercise from 0.031 ± 0.010 to 0.067 ± 0.013%·h^–1^ and 0.030 ± 0.008 to 0.063 ± 0.010%·h^–1^, respectively (Time: all *P* < 0.05; Time × Leg: *P* = 0.002), with no differences between cheese and milk protein ingestion (Time × Group: both *P* > 0.05).

**Conclusion:**

Cheese ingestion increases muscle protein synthesis rates both at rest and during recovery from exercise. The postprandial muscle protein synthetic response to the ingestion of cheese or milk protein does not differ when 30 g protein is ingested at rest or during recovery from exercise in healthy, young males.

## Introduction

It has been well-established that dietary protein ingestion increases muscle protein synthesis rates (1–3). Exercise performed prior to protein ingestion further enhances the muscle protein synthetic response to protein ingestion (4, 5). Furthermore, it has been shown that the anabolic response following protein ingestion is modulated by the amount of protein (6, 7), the type of protein (8–10), as well as protein processing prior to ingestion (11). The capacity of a protein to stimulate postprandial muscle protein synthesis is largely determined by its protein digestion and amino acid absorption kinetics (8, 9, 12) and amino acid composition (9, 10, 13), with leucine content being of particular relevance (14, 15).

Milk protein, and its constituents whey and casein protein, are the most studied proteins for their capacity to stimulate muscle protein synthesis. Numerous studies have reported rapid protein digestion and amino acid absorption (9, 16) and substantial increases in muscle protein synthesis rates (1, 2, 17–21) following ingestion of different milk protein isolates and concentrates both at rest and during recovery from exercise. However, far less information is available on the anabolic response following ingestion of whole-food dairy protein sources. In a normal eating pattern, protein is mainly consumed in the form of protein-rich whole foods as opposed to milk protein isolates or concentrates (22). Whole-food products contain multiple nutrients incorporated in a complex structure that can modulate food digestion and subsequent nutrient absorption, which is referred to as the food matrix (23). Consequently, this food matrix can strongly affect the metabolic impact of consumed nutrients. In the present study, we aim to contribute to the growing interest in understanding the impact of the consumption of whole foods, as opposed to their isolated nutrients, on postprandial nutrient bioavailability and functionality (23, 24).

Cheese is a nutrient-dense whole food commonly consumed as part of the Western diet (25). Cheese is produced from dairy milk in a multi-stage and variable process that involves fermentation, coagulation, drying, and ripening. Since most whey is removed from milk following the coagulation of casein, cheese contains mainly the casein fraction of milk protein (26). As a whole food, cheese is protein dense, with 58% of its energy content (energy percent [En%]) being provided by protein. Consequently, we hypothesized that ingestion of a meal-sized amount of cheese increases circulating plasma amino acid concentrations and subsequently stimulates muscle protein synthesis. Recent work has reported substantial differences in postprandial circulating amino acid concentrations following ingestion of dairy protein isolates, concentrates, and various dairy whole foods (27, 28). Whether such differences in postprandial plasma amino acid concentrations modulate the capacity to stimulate postprandial muscle protein synthesis remains to be established (28). The food matrix of cheese and the greater amount of casein protein in cheese when compared with milk protein (26) could theoretically result in an attenuated postprandial rise in circulating essential amino acid (EAA) concentrations, and plasma leucine concentrations in particular. Therefore, we hypothesized that ingestion of a meal-sized amount of protein provided as cheese will result in a lesser postprandial rise in muscle protein synthesis rates when compared to the ingestion of an equivalent amount of milk protein at rest and/or during recovery from exercise.

In this study, we compared the impact of ingesting 30 g protein provided in the form of cheese or milk protein concentrate on postprandial muscle protein synthesis rates in vivo in 20 healthy, young males. Combining the intravenous infusion of L-[*ring*-^13^C_6_]-phenylalanine with repeated blood sampling and muscle biopsy collection allowed us to assess postprandial plasma amino acid profiles and muscle protein synthesis rates following the ingestion of cheese or milk protein both at rest and during recovery from exercise.

## Methods

### Subjects

Twenty healthy, young males (age: 25 ± 4 y; BMI: 23.0 ± 2.6 kg·m^–2^) were selected to participate in this study. **Table 1** details the characteristics of the subjects. The study was conducted between August 2019 and December 2020 at Maastricht University, Maastricht, The Netherlands (for the CONSORT flow diagram, please see **Supplemental Figure 1**). All subjects were informed of the nature and possible risks of the experimental procedures, before providing written informed consent. This study was approved by the Medical Ethical Committee of the Academic Hospital Maastricht/Maastricht University and performed conforming to the principles outlined in the Declaration of Helsinki for the use of human subjects and tissue. This study was registered at www.trialregister.nl as NL7941.

**TABLE 1:** Subjects’ characteristics^1^

	MILK	CHEESE
	(*n* = 10)	(*n* = 10)
Age, y	25 ± 5	25 ± 4
Body mass, kg	75.1 ± 8.7	70.4 ± 6.3
Lean body mass, kg	55.2 ± 4.9	54.1 ± 5.5
Appendicular lean mass, kg	25.8 ± 2.4	25.0 ± 2.5
Body fat, %	23.0 ± 6.7	20.6 ± 3.3
Height, m	1.80 ± 0.07	1.76 ± 0.06
BMI, kg·m^–2^	23.2 ± 3.2	22.8 ± 1.9
1-RM leg press rest leg, kg	117 ± 20	119 ± 21
1-RM leg press exercise leg, kg	117 ± 15	126 ± 19
1-RM leg extension rest leg, kg	56 ± 8	56 ± 8
1-RM leg extension exercise leg, kg	54 ± 9	54 ± 5

1 All values represent means ± SDs, *n* = 10 per group. 1-RM, 1-repetition maximum; CHEESE, 30 g cheese protein; MILK, 30 g milk protein.

### Pretesting

Volunteers aged between 18 and 35 y with a BMI between 18.5 and 30.0 underwent screening to assess body mass, height, and body composition (by DXA; Discovery A; Hologic). The participants were deemed healthy and eligible to participate based upon their response to a medical questionnaire and screening results. Participants were excluded if they were habitual smokers, using medication that affected protein metabolism, intolerant to the investigated proteins, or participated in an amino acid tracer trial 1 y prior to inclusion. After initial screening, participants were familiarized with the exercise testing protocol and exercise equipment. Participants underwent estimates of unilateral 1 repetition maximum (1-RM) leg strength on the supine leg press (Technogym BV) and seated leg extension (Technogym BV) for both legs separately. As a warm-up, participants performed 2 sets of 10 repetitions with submaximal weights. Next, the participants performed single repetitions with incremental weights for every repetition, until failure occurred. Participants switched legs between every attempt and a rest period of ≥2 min was taken for each leg. The 1-RM was usually reached between 5 and 7 attempts. The experimental test day was scheduled ≥1 wk after pretesting.

### Study design

In this randomized, parallel-group intervention trial, subjects were randomly assigned to consume 30 g protein provided as cheese (CHEESE; *n* = 10) or milk protein concentrate (MILK; *n* = 10). A block randomization with blocks of 4 participants was performed before the study by one of the researchers using a computerized random-number generator. Within each treatment group the exercise leg was randomly assigned. The participants were assigned to the intervention groups at the start of the experimental test day. The nature of the interventions did not allow blinding of the researchers or the participants. However, research technicians performing the final analyses of samples were blinded to the intervention allocation.

### Diet and physical activity

All subjects refrained from any sort of strenuous physical activity or exercise 3 d prior to the trial and kept their diet as consistent as possible 2 d prior to the experiment. On the evening before the experimental trial at 20:00, all subjects consumed the same standardized meal (1.71 MJ/405 kcal) providing 22 En% protein, 53 En% carbohydrate, and 26 En% fat, after which they fasted.

### Study protocol

**FIGURE 1:**
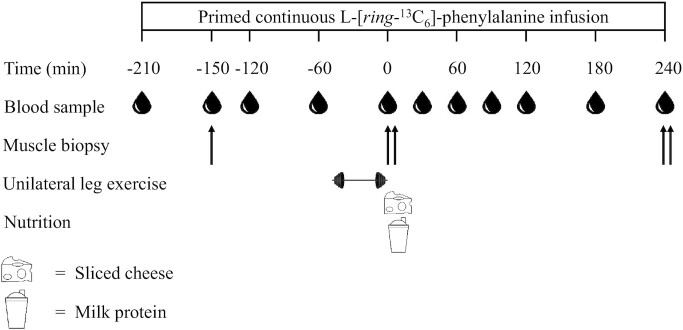
Schematic overview of the protocol. Participants performed unilateral leg exercise before ingesting 30 g protein provided as cheese or milk protein concentrate.

**Figure 1** depicts a schematic overview of the protocol. At 07:45 , following an overnight fast, subjects arrived at the laboratory by car or public transport. A catheter was inserted into an antecubital vein for stable isotope labeled amino acid infusion. A second catheter was inserted into a dorsal hand vein of the contralateral arm and placed in a hot box (60°C) for arterialized blood sampling (29). After taking a baseline blood sample, the plasma amino acid pool was primed with a single dose of L-[*ring*-^13^C_6_]-phenylalanine (2.25 μmol·kg^–1^), after which a continuous intravenous L-[*ring*-^13^C_6_]-phenylalanine (0.05 μmol·kg^–1^·min^–1^) infusion was initiated (*t* = –210 min). Subsequently, the subjects rested in a supine position for 60 min after which a blood sample was taken together with a muscle biopsy from the *M. vastus lateralis* from the assigned rested leg (REST; *t* = –150 min). After taking 2 more blood samples at *t* = –120 and –60 min, the single-legged exercise session was initiated (*t* = –50 min). The exercise session consisted of 6 sets on both the leg press and leg extension machine. The first and second set functioned as a warm-up (20 repetitions at 25% 1-RM and 10 repetitions at 50% 1-RM), followed by 3 sets of ∼8–10 repetitions (at 80% 1-RM) and the last set was performed at the same resistance until volitional fatigue occurred. Subjects rested for 2 min between all sets and exercises and were verbally encouraged to complete the protocol.

Immediately following the exercise, a blood sample was taken and a muscle biopsy sample was collected from both the exercised (EXERCISE) and the rested (REST) leg. Immediately thereafter 30 g protein, provided as cheese or milk protein concentrate, was consumed (*t =* 0 min). Further arterialized blood samples were collected at *t* = 30, 60, 90,120, 180, and 240 min. Additional muscle biopsies were taken at *t* = 240 min from both legs to determine postprandial mixed muscle protein synthesis rates at rest and during recovery from exercise. Blood samples were collected in tubes containing EDTA and centrifuged at 1000 × *g* at 4°C for 10 min. Aliquots of plasma were frozen in liquid nitrogen and stored at –80°C until further analysis. Muscle biopsies were taken from the middle region of the *M. vastus lateralis*, 15 cm above the patella and 3 cm below entry through the fascia, using a modified Bergström needle (30). Muscle samples were dissected carefully, freed from any visible fat, immediately frozen in liquid nitrogen, and stored at –80°C until further analysis.

### Preparation of tracer

The stable isotope tracer L-[*ring*-^13^C_6_]-phenylalanine was purchased from Cambridge Isotopes and dissolved in 0.9% saline before infusion (Apotheek A15).

### Nutrition

Subjects ingested 30 g protein provided as cheese or milk protein concentrate with water (matched for total water volume, with 2.5% L-[*ring*-^13^C_6_]-phenylalanine added). The cheese was provided as 103 g sliced cheese (Milner jong 30+, FrieslandCampina) served with 250 mL water. The milk protein represented 37 g milk protein concentrate (REFIT MPC80, FrieslandCampina) dissolved into 300 mL water. Protein quantity was matched to provide 30 g protein according to protein content as described by the manufacturer. The sliced cheese provided in total 1.2 MJ/292 kcal as 58 En% (30 g) protein,  0 En% (0 g) carbohydrate, and 42 En% (19 g) fat. The milk protein provided in total 0.6 MJ/142 kcal as 91 En% (30 g) protein, 6 En% (2 g) carbohydrate, and 4 En% (1 g) fat. Amino acid compositions of the cheese and milk protein concentrate were measured in triplicate (ultra-performance liquid chromatograph MS [UPLC-MS]; ACQUITY UPLC H-Class with QDa; Waters) and are shown in **Table 2**.

**TABLE 2:** Amino acid content of 30 g protein provided as milk protein concentrate or cheese^1^

Amino acid	MILK	CHEESE
Alanine, g	0.9	0.6
Arginine, g	0.8	0.9
Aspartic acid, g	1.6	1.6
Glutamic acid, g	5.6	5.0
Glycine, g	0.5	0.5
Histidine, g	0.6	0.6
Isoleucine, g	0.9	0.9
Leucine, g	2.6	2.4
Lysine, g	2.0	2.0
Methionine, g	0.8	0.7
Phenylalanine, g	1.3	1.2
Proline, g	3.1	2.1
Serine, g	1.2	1.2
Threonine, g	1.0	0.8
Tyrosine, g	1.4	1.4
Valine, g	1.1	1.2
*EAA*, g	*10.3*	*9.8*
*NEAA*, g	*15.2*	*13.4*
*TAA*, g	*25.6*	*23.2*

1 Values represent means of the triplicate measurements. EAA, essential amino acids; NEAA, nonessential amino acids; TAA, total amino acids. Note: asparagine, cysteine, glutamine, and tryptophan were not measured.

### Plasma and muscle tissue analyses

Plasma glucose and insulin concentrations were measured using commercially available kits (GLUC3, Roche, Ref: 05,168,791 190, and Immunologic, Roche, Ref: 12,017,547 122, respectively). Plasma amino acid concentrations were quantified using UPLC-MS as described previously (1). Plasma phenylalanine concentrations and L-[*ring*-^13^C_6_]-phenylalanine enrichments were determined by GC-MS (Agilent 7890A GC/5975C; MSD) as described previously (17). For measurement of muscle protein-bound L-[*ring*-^13^C_6_]-phenylalanine enrichments, muscle protein was first extracted as described previously (2). Next, muscle protein-bound L-[*ring*-^13^C_6_]-phenylalanine enrichments were measured using GC-isotope ratio MS (GC-IRMS) as described previously (17).

### Calculations

The incremental AUC (iAUC) were calculated using the trapezoid rule. Mixed muscle protein fractional synthetic rates (FSRs) were calculated by using the standard precursor-product equation, as follows:

(1) \begin{eqnarray*} FSR = \frac{{\Delta {E_p}}}{{{E_{precursor}}t}} \cdot 100 \end{eqnarray*}

Δ*Ep* is the increment in mixed muscle protein-bound L-[*ring*-^13^C_6_]-phenylalanine enrichment after an incorporation period, *E*_precursor_ is the weighted mean plasma L-[*ring*-^13^C_6_]-phenylalanine enrichment during that incorporation period, and *t* is the incorporation period (h). Weighted mean plasma L-[*ring*-^13^C_6_]-phenylalanine enrichments were calculated by taking the average enrichment between all consecutive time points and correcting for the time between these sampling time points. The weighted mean plasma L-[*ring*-^13^C_6_]-phenylalanine precursor pool is preferred in this setting, because the more frequent sampling time points allow for a more accurate correction of the transient changes in precursor pool enrichments over time when compared to the muscle intracellular free amino acid pool (31). For basal FSR calculations, data derived from the muscle biopsy samples from the rested leg at *t* = –150 min and muscle tissue samples collected from both legs at *t* = 0 min were used. For postprandial FSR calculations, data derived from muscle biopsy samples collected at *t* = 0 and 240 min were used for both the rested and the exercised leg.

### Statistical analysis

All data are expressed as means ± SDs and there were no missing data. Postprandial FSRs were the primary outcome of this study. Secondary outcomes included basal FSRs, plasma glucose, plasma insulin, plasma amino acids, iAUCs, plasma enrichments, dietary intake outcomes, and participant characteristics. No outcomes were classified as exploratory. Normality of the data was checked using the Shapiro–Wilk test and sphericity was checked using Mauchly's test of sphericity and in case that test was significant, the Greenhouse–Geisser corrected values were used for interpretation of the repeated measures ANOVA. Peak plasma concentrations and iAUCs were compared between groups using the independent-samples t-test. For time-dependent plasma variables, repeated-measures ANOVA with group (MILK or CHEESE) as a between-subjects factor, and time as a within-subjects factor were used. For time-dependent muscle variables, repeated-measures ANOVA with group (MILK or CHEESE) as a between-subjects factor, and both time (basal compared with postprandial) and leg (exercise compared with rest) as a within-subjects factor were used. In case of significant interactions, separate analyses were performed as appropriate (i.e. within each group or within legs, as well as between groups or between legs for every time period separately). In the case of significant time effects, Bonferroni post hoc analyses were performed to locate the differences for plasma variables. Effect size estimates were calculated for the primary FSR outcomes (partial *η^2^*) and important secondary outcomes such as postprandial plasma amino acid concentrations (partial *η^2^*) and iAUCs (Cohen's *d*). Significance was set at *P* ≤0.05 and all statistical testing was performed 2-sided. Calculations were performed using SPSS (version 25.0, IBM Corp.) Sample size was calculated with differences in postprandial muscle FSR as the primary outcome measure. Based on studies comparing muscle protein turnover between protein sources at rest and after exercise (2, 7, 9), we used a 20% difference between protein sources in muscle FSR (i.e. 0.040 compared with 0.048%·h^–1^) with an SD of ∼16% (i.e. 0.006%·h^–1^) to calculate the required sample size. Using a power of 80%, a significance level of 0.05, and a 10% dropout, the final number of participants was calculated as *n* = 10 per group.

## Results

### Participants’ characteristics and dietary intake records

Participants’ characteristics are shown in Table 1. Daily habitual food intake provided 9.8 ± 2.9 and 7.9 ± 2.4 MJ/d in the 2 d prior to the experimental trial in the CHEESE and MILK group. Carbohydrate, fat, and protein provided 45 ± 10 and 47 ± 6%, 35 ± 8 and 33 ± 5%, and 17 ± 4 and 17 ± 4% of the energy consumed in the CHEESE and MILK group. Protein intake averaged 1.4 ± 0.4 and 1.1 ± 0.5 g per kg body mass per day in the CHEESE and MILK group, respectively.

### Plasma glucose and insulin concentrations

**FIGURE 2:**
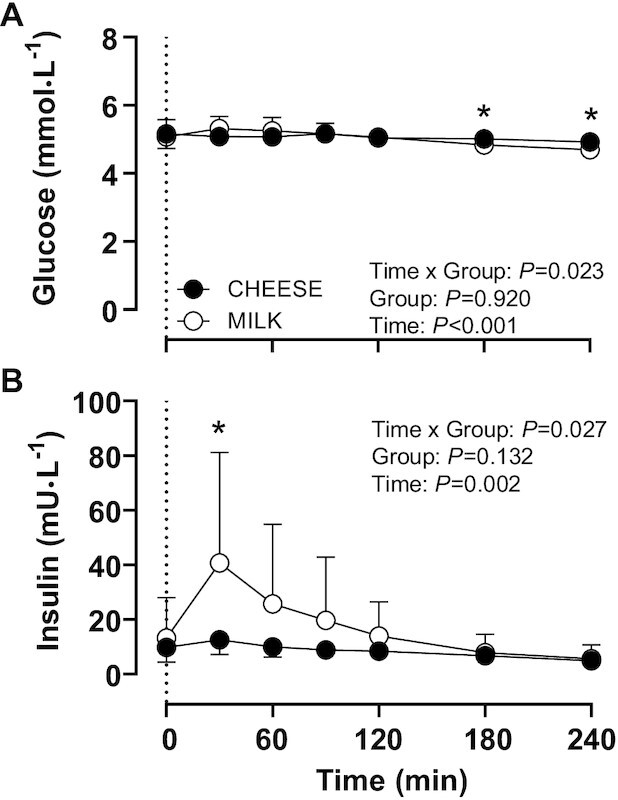
Plasma glucose (A) and insulin (B) concentrations following the ingestion of 30 g protein provided as cheese (CHEESE; *n* = 10) or milk protein concentrate (MILK; *n* = 10) during 4 h of recovery from a single bout of unilateral exercise in healthy, young males. The dotted line represents the time of protein ingestion. Values represent means ± SDs. Data were analyzed using repeated measures (Time x Group) ANOVA and separate analysis were performed when a significant interaction was detected. Bonferroni posthoc testing was used to detect differences between time points. *, MILK significantly different from CHEESE.

For both plasma glucose and insulin concentrations a Time × Group interaction was observed (**Figure 2**; *P* < 0.05). Plasma glucose concentrations did not change significantly following protein ingestion in both groups (*P* > 0.05). MILK ingestion significantly increased plasma insulin concentrations by 208%, which remained elevated for 60 min after protein ingestion (*P* < 0.05). No significant changes in plasma insulin concentrations were observed following CHEESE ingestion (*P* > 0.05).

### Plasma amino acid concentrations

**FIGURE 3:**
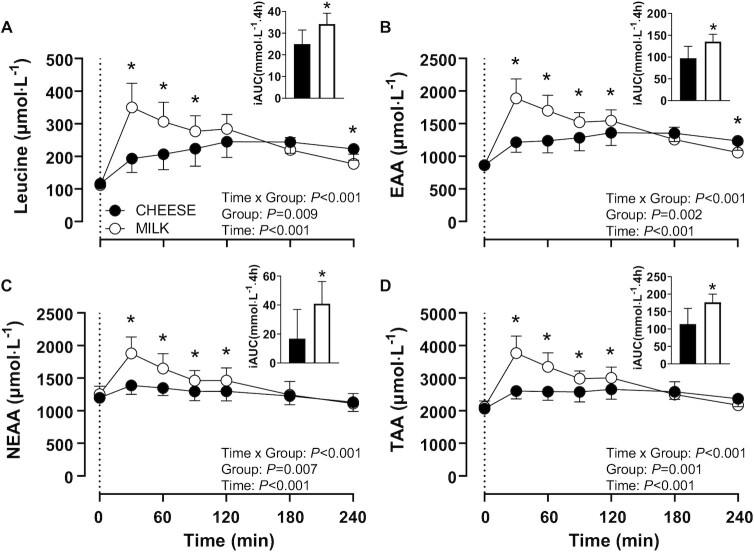
Plasma leucine (A), total essential amino acid (B; EAA), total nonessential amino acid (C; NEAA), and total amino acid (D; TAA) concentrations and incremental AUC (iAUC) concentrations following the ingestion of 30 g protein provided as cheese (CHEESE; *n* = 10) or milk protein concentrate (MILK; *n* = 10) during 4 h of recovery from a single bout of unilateral exercise in healthy, young males. The dotted line represents the time of protein ingestion. Values represent means ± SDs. Data were analyzed using repeated measures (Time × Group) ANOVA and separate analysis were performed when a significant interaction was detected. Bonferroni posthoc testing was used to detect differences between time points. *, MILK significantly different from CHEESE.

Figures for individual plasma amino acid concentrations are provided in **Supplemental Figures 2–4**. For plasma leucine, EAA, nonessential amino acid (NEAA), and total amino acid (TAA) concentrations, a main effect of Time, Group, and a Time × Group interaction were observed (**Figure 3**; *P* < 0.05). Plasma leucine and EAA concentrations (Figure 3A and 3B) increased following both CHEESE and MILK ingestion but peaked 38 and 34% higher for MILK when compared with CHEESE, respectively (both *P* < 0.001; *η^2^_p_* = 0.54 and 0.54, respectively). Following protein ingestion, plasma leucine and EAA concentrations remained elevated for the full postprandial period, with higher values near the end of the postprandial period following CHEESE compared with MILK ingestion (*P* < 0.05). Plasma NEAA concentrations (Figure 3C) only increased following MILK ingestion (*P* < 0.05), with peak values being 35% higher in MILK compared with CHEESE (*P* < 0.001; *η^2^_p_* = 0.40). Plasma TAA concentrations (Figure 3D) increased following both CHEESE and MILK ingestion, with peak values 38% higher for MILK when compared with CHEESE (*P* < 0.001; *η^2^_p_* = 0.50). In the MILK group, plasma TAA concentrations returned to baseline values within 4 h, whereas in the CHEESE group plasma TAA concentrations remained elevated. For plasma leucine, EAA, NEAA, and TAA concentrations, increments of amino acid availability over the 4-h postprandial period, as expressed by the iAUC, were higher for MILK when compared with CHEESE (all *P* < 0.05; *d* = 1.59, 1.69, 1.39, and 1.76, respectively).

**FIGURE 4:**
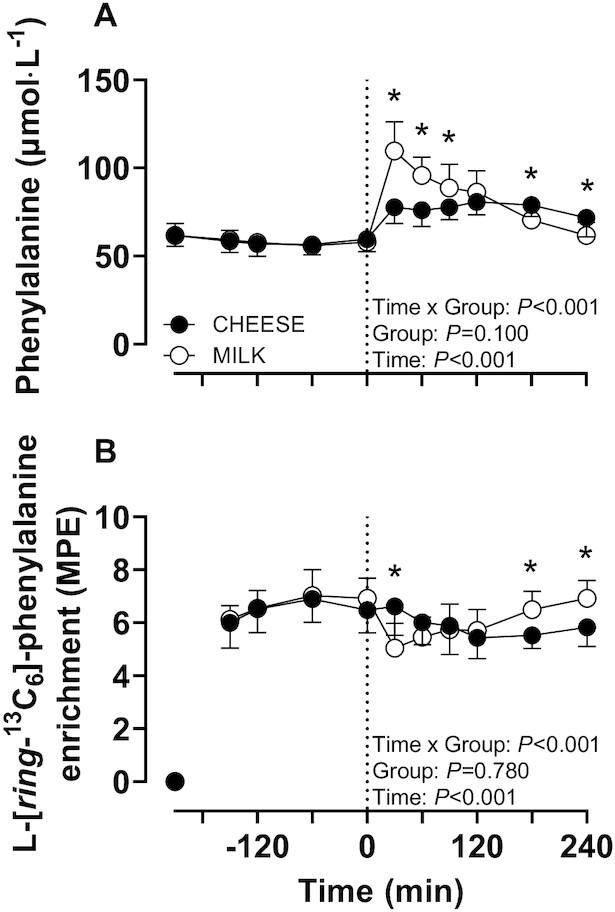
Plasma phenylalanine concentrations (A) and plasma L-[*ring*-^13^C_6_]-phenylalanine enrichments (B) following the ingestion of 30 g protein provided as cheese (CHEESE; *n* = 10) or milk protein concentrate (MILK; *n* = 10) during 4 h of recovery from a single bout of unilateral exercise in healthy, young males. The dotted line represents the time of protein ingestion. Values represent means ± SDs. Data were analyzed using repeated measures (Time × Group) ANOVA and separate analysis were performed when a significant interaction was detected. Bonferroni posthoc testing was used to detect differences between time points. *, MILK significantly different from CHEESE; MPE, mole percent excess.

For plasma phenylalanine concentrations and L-[*ring*-^13^C_6_]-phenylalanine enrichments, a main effect of Time and a Time × Group interaction were observed (**Figure 4**; *P* < 0.001). Plasma phenylalanine concentrations increased following both CHEESE and MILK ingestion (*P* < 0.05), and remained elevated for the full postprandial period for CHEESE but not for MILK. In the basal period (*t* = –120 until 0 min), plasma L-[*ring*-^13^C_6_]-phenylalanine enrichments were stable and did not differ between groups (*P* > 0.05). Following protein ingestion, plasma L-[*ring*-^13^C_6_]-phenylalanine enrichments decreased in the MILK group, with an attenuated decline in the CHEESE group (*P* < 0.05). Plasma L-[*ring*-^13^C_6_]-phenylalanine enrichments during the 4-h postprandial period averaged 5.8 and 6.0 mole percent excess (MPE) for CHEESE and MILK, respectively, and did not differ between groups (*P* = 0.54).

### Muscle protein synthesis rates

**FIGURE 5:**
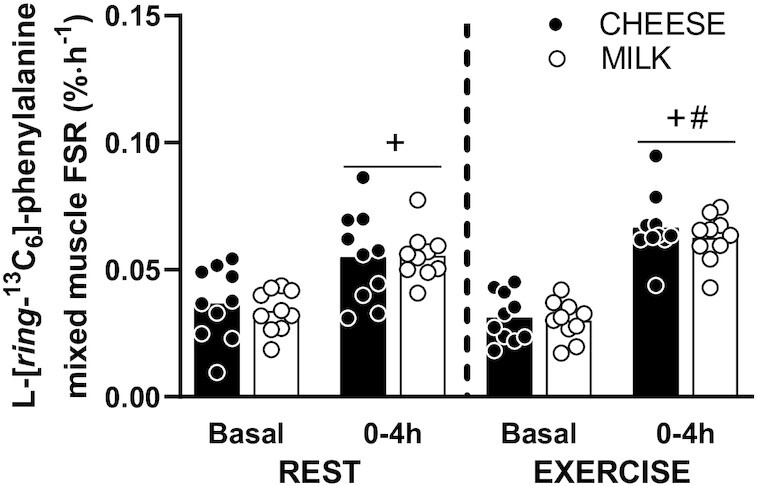
Mixed muscle protein fractional synthesis rates (FSR) following the ingestion of 30 g protein provided as cheese (CHEESE; *n* = 10) or milk protein concentrate (MILK; *n* = 10) in both the rested (REST) and exercised (EXERCISE) leg in healthy, young males. Bars represent means and dots represent individual values. Data were analyzed using repeated measures (Time × Leg × Group) ANOVA, and separate analysis were performed when a significant interaction was detected. There was no significant Time × Leg × Group interaction (*P* = 0.42). A significant Time × Leg interaction was observed (*P* = 0.002). +, Postprandial FSR significantly different from basal values; #, Postprandial FSR in the EXERCISE leg significantly different from postprandial FSR in the REST leg.

Muscle protein synthesis rates did not show a significant Time × Leg × Group interaction (**Figure 5**; *P* = 0.42; *η^2^_p_* = 0.04), but did show a significant Time × Leg interaction (*P* = 0.002; *η^2^_p_* = 0.43) indicating muscle protein synthesis rates increased more in the EXERCISE when compared to the REST leg. In the REST leg, muscle protein synthesis rates (Figure 5) increased from basal to postprandial (0–4 h) following both CHEESE (from 0.037 ± 0.014 to 0.055 ± 0.018%·h^–1^) and MILK (from 0.034 ± 0.008 to 0.056 ± 0.010%·h^–1^) ingestion (Time: *P* < 0.001; *η^2^_p_* = 0.61), with no differences between groups (Time x Group: *P* = 0.64; *η^2^_p_* = 0.01). In the EXERCISE leg, muscle protein synthesis rates (Figure 5) increased from basal to postprandial (0–4 h) following both CHEESE (from 0.031 ± 0.010 to 0.067 ± 0.013%·h^–1^) and MILK (from 0.030 ± 0.008 to 0.063 ± 0.009%·h^–1^) ingestion (Time: *P* < 0.001; *η^2^_p_* = 0.87), with no differences between groups (Time × Group: *P* = 0.66; *η^2^_p_* = 0.01). Postprandial muscle protein synthesis rates were higher in the EXERCISE compared with the REST leg (Leg: *P* = 0.006; *η^2^_p_* = 0.35), with no differences between groups (Leg × Group: *P* = 0.44; *η^2^_p_* = 0.03).

## Discussion

In the present study we observed a significant increase in circulating amino acid concentrations following the ingestion of 30 g protein provided either as cheese or milk protein. The early postprandial rise in circulating amino acids was attenuated, but subsequently more sustained following cheese compared with milk protein ingestion. Muscle protein synthesis rates increased substantially following both cheese and milk protein ingestion, with greater increases observed when exercise was performed prior to protein ingestion. No differences were observed in postprandial muscle protein synthesis rates at rest or during recovery from exercise following cheese compared with milk protein ingestion.

Ingestion of 30 g protein provided as cheese was followed by a robust increase in plasma amino acid concentrations (Figure 3). The postprandial increment in TAA, EAA, and NEAA was attenuated following the ingestion of cheese when compared to the ingestion of an equivalent amount of protein provided as milk protein concentrate. In agreement, the postprandial increase in plasma amino acid availability (iAUC) was substantially less following cheese when compared with milk protein ingestion. These findings seem to be in line with previous work (28) reporting a more delayed increase in plasma amino acid availability following cheddar cheese compared with milk ingestion. Our data further support the understanding that the food matrix in which protein is provided strongly affects protein digestion and amino acid absorption, and modulates the subsequent postprandial plasma amino acid profile. In the present study we can only speculate on the various factors that may have contributed to the attenuated, but more sustained, postprandial rise in circulating amino acids following cheese compared with milk protein ingestion. The lower leucine content in cheese when compared with milk protein (Table 2), could partially explain the lower circulating postprandial leucine concentrations. However, it seems evident that the higher energy (32–34), fat (35), and casein (9, 11) content of cheese are responsible for a delay in gastric emptying and subsequent attenuation of protein digestion and amino acid absorption. Furthermore, the processing of milk to produce cheese may have also impacted protein structure and function that could modulate protein digestion and amino acid absorption kinetics of the native protein (36–38).

Protein ingestion and the subsequent rise in circulating (essential) amino acids have been shown to increase muscle protein synthesis rates (1, 2, 39–41). In agreement, we observed a strong increase in muscle protein synthesis rates following the ingestion of 30 g protein, provided either as cheese or milk protein, respectively (Figure 5). These data extend on previous work on postprandial muscle protein synthesis rates observed following the ingestion of 30 g milk protein (1, 2, 17, 19) and add a comparison with postprandial muscle protein synthesis rates following ingestion of an isonitrogenous amount of a dairy whole food. Despite the substantial differences in postprandial plasma amino acid concentrations (Figure 3), we did not observe any differences in postprandial muscle protein synthesis rates following cheese versus milk protein ingestion. Clearly, the cheese matrix did not compromise the muscle protein synthetic response, despite the attenuated postprandial rise in circulating plasma amino acid concentrations. We speculate that this may also be attributed to the more sustained elevation of plasma amino acid availability following cheese ingestion when compared with the milk protein concentrate.

Exercise performed prior to food ingestion augments the postprandial increase in muscle protein synthesis rates and allows more of the protein-derived amino acids to be used for de novo muscle protein synthesis (2, 4, 42). In the present study, participants performed a single-legged resistance-type exercise session prior to protein intake, allowing us to assess postprandial muscle protein synthesis rates both at rest as well as during recovery from exercise. In the exercised leg muscle protein synthesis rates increased more than in the rested leg. However, no differences were observed in postexercise muscle protein synthesis rates following the ingestion of cheese versus milk protein, respectively (Figure 5). So, despite apparent differences in postprandial plasma amino acid availability following cheese versus milk protein ingestion, no differences were observed in muscle protein synthesis rates at rest or during recovery from exercise.

Obviously, by including multiple outcomes (i.e. multiple plasma AA parameters and muscle FSRs), the overall chance of making a type I error is increased. We did not correct for this as our main aim was to detect any potential differences between treatments, and further correction would increase chances for a type II error. Alternatively, we provide effects size estimates to further support the magnitude of treatment differences. In doing so, the present study shows that the food matrix in which protein is ingested can strongly affect postprandial protein handling, with a blunted but more sustained rise in circulating amino acid concentrations following cheese compared with milk protein ingestion. When ingesting 30 g of protein, the postprandial muscle protein synthetic response was not compromised by the whole-food matrix of cheese. In the current study, we provided our participants with 30 g protein, which is more than the 20 g protein that has previously been reported to maximize muscle protein synthesis rates (6, 7). We can only speculate whether differences in the muscle protein synthetic response to cheese versus milk protein may become apparent when (much) lesser amounts of protein are provided. The present data extend on previous work on the anabolic properties of dairy protein isolates and concentrates, and suggest that previous findings on the postprandial stimulation of muscle protein synthesis following the ingestion of ample amounts of dairy protein concentrates also apply to the consumption of dairy whole foods. Clearly, (dairy) whole foods, that are typically consumed as part of our diet, provide strong stimuli for muscle protein synthesis. These observations were evident both at rest and during recovery from exercise, showing that the use of protein isolates or concentrates does not necessarily provide any surplus benefits over the consumption of protein-dense whole foods when trying to augment muscle mass accretion. Whether the same holds true for healthy older or more clinically compromised populations remains to be established. The prevalence of anabolic resistance (43) may compromise the capacity to stimulate muscle protein synthesis following a more moderate postprandial rise in circulating amino acids after the ingestion of a protein-dense whole food versus its extracted protein isolate or concentrate. Future work will be needed to translate the extensive body of research on postprandial protein metabolism in a setting where whole foods and/or mixed meals are being consumed.

In conclusion, cheese ingestion increases muscle protein synthesis rates both at rest and during recovery from exercise. There were no differences in the postprandial rise in muscle protein synthesis rates following ingestion of 30 g protein provided either as cheese or as milk protein at rest or during recovery from exercise in healthy, young males. Protein-dense whole foods can be applied as effectively as protein concentrates to support muscle protein accretion both at rest and during recovery from exercise.

## Supplementary Material

nxac007_Supplemental_File

## Data Availability

Data described in the article, code book, and analytical code will be made available upon request pending application and approval.

## Notes

Supporter by The Dutch Dairy Association.

Author disclosures: LJCvL and LBV have received research grants, consulting fees, speaking honoraria, or a combination of these, from Friesland Campina and Arla Food Ingredients. All other authors report no conflicts of interest.

Supplemental Figures 1–4 are available from the “Supplementary data” link in the online posting of the article and from the same link in the online table of contents at https://academic.oup.com/jn/.

Abbreviations used: CHEESE, 30 g protein from cheese; EAA, sum of essential amino acids; En%, energy%; EXERCISE, exercised leg; FSR, fractional synthesis rate; iAUC, incremental AUC; MILK, 30 g milk protein; NEAA, sum of nonessential amino acids; REST, rested leg; TAA, sum of total amino acids; UPLC-MS, ultra-performance liquid chromatograph MS; 1-RM, 1 repetition maximum.
