# Supplementary material for: Cheese Ingestion Increases Muscle Protein Synthesis Rates Both at Rest and During Recovery from Exercise in Healthy, Young Males: A Randomized Parallel-Group Trial
Source: J Nutr. 2022 Jan 10;152(4):1022–30. doi: 10.1093/jn/nxac007 (PMC8971000; doi:10.1093/jn/nxac007)
Supplement: nxac007_Supplemental_File [file nxac007_supplemental_file.pdf]

This is the online supplementary material to the manuscript: *Cheese ingestion increases muscle protein synthesis rates both at rest and during recovery from exercise in healthy, young males: A randomized parallel-group trial* by Wesley J.H. Hermans, Cas J. Fuchs, Floris K. Hendriks, Lisanne H.P. Houben, Joan M. Senden, Lex B. Verdijk, and Luc J.C. van Loon.

METHODS

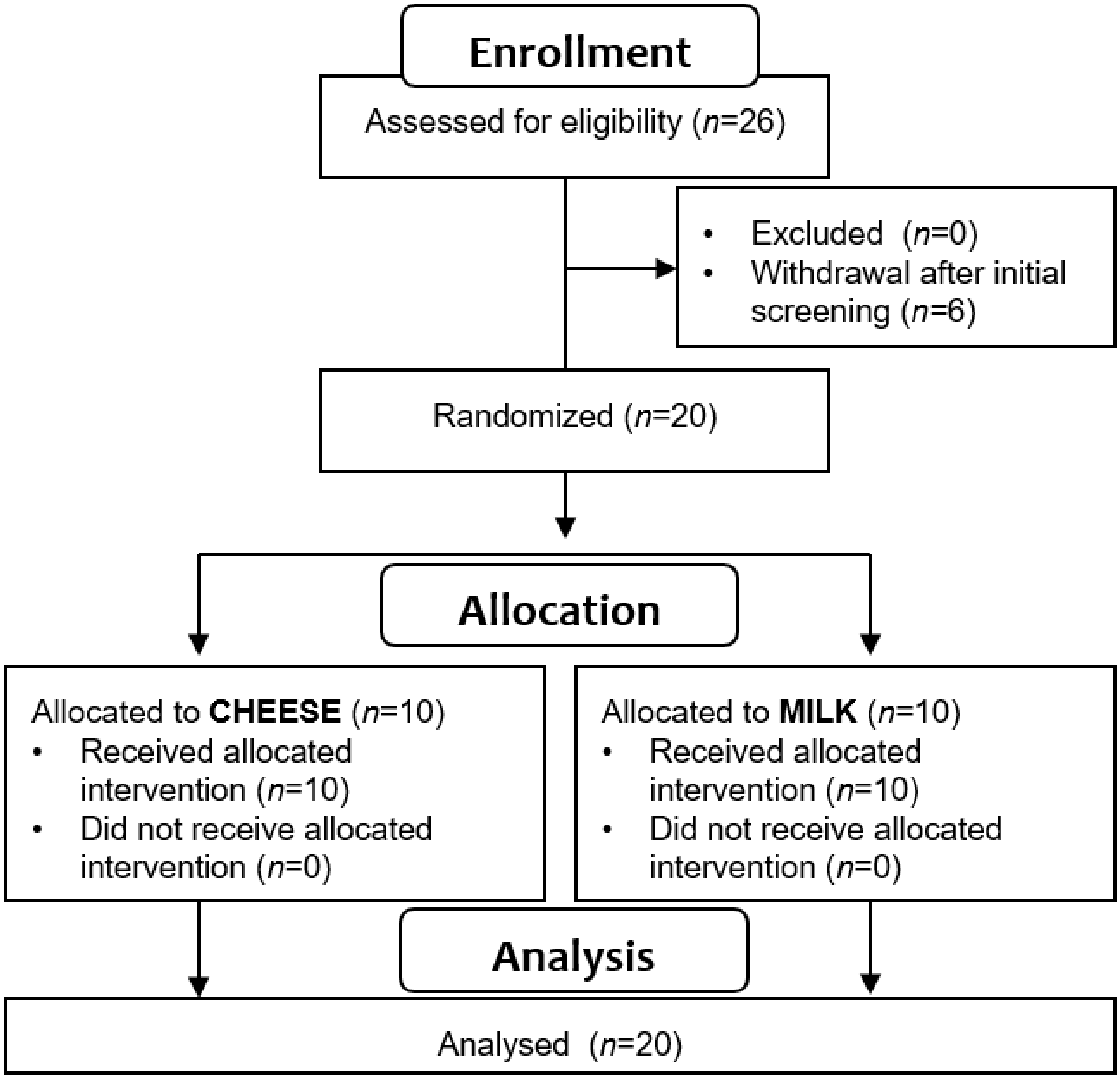

Supplemental figure 1 Consolidated Standards of Reporting Trials (CONSORT) flow diagram.

RESULTS

Figures below describe plasma amino acid concentrations quantified using ultra-performance liquid chromatograph mass spectrometry (UPLC-MS; ACQUITY UPLC H-Class with QDa; Waters, Saint-Quentin, France) as described previously (1).

For plasma arginine, asparagine, aspartic acid, glutamic acid, glycine, histidine, isoleucine, leucine, lysine, methionine, phenylalanine, proline, serine, threonine, tryptophan, tyrosine, valine, BCAA, EAA, NEAA, and TAA a significant Time x Group interaction was observed (all $P<0.05$), whereas for cysteine this interaction was not present ($P>0.05$).

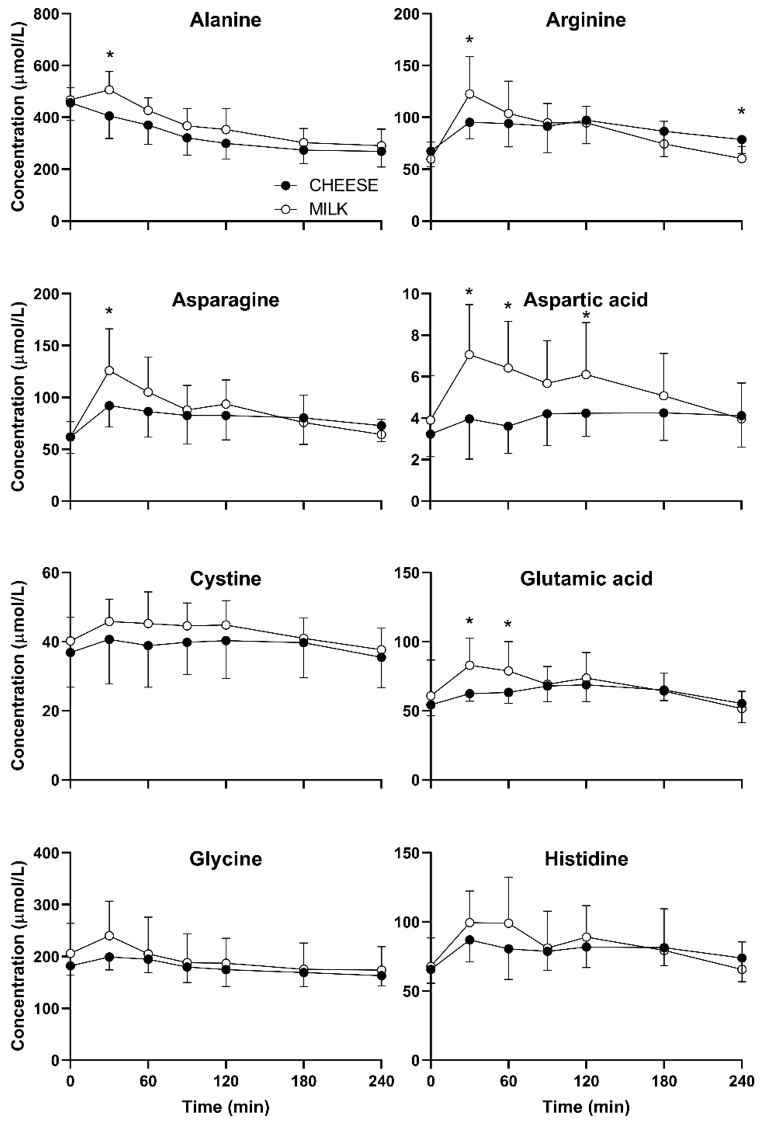

Supplemental figure 2 Plasma amino acid concentrations following ingestion of 30 g protein provided as cheese (CHEESE; *n*=10) or milk protein concentrate (MILK; *n*=10) during 4 h of recovery from a single bout of unilateral exercise in healthy, young males. t=0 min represents the time of protein ingestion. Values represent means±SD. Data were analyzed using repeated measures (Time x Group) ANOVA and separate analysis were performed when a significant interaction was detected. Bonferroni post hoc testing was used to detect differences between timepoints. *, MILK significantly different from CHEESE.

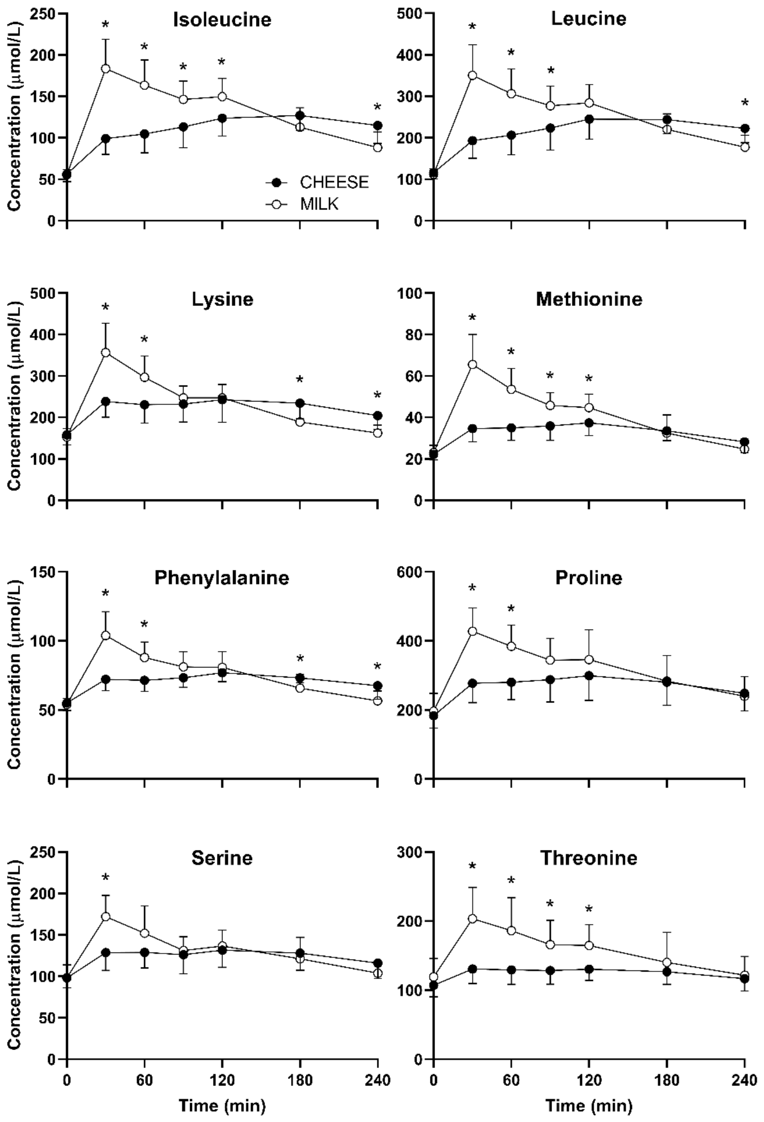

Supplemental figure 3 Plasma amino acid concentrations following ingestion of 30 g protein provided as cheese (CHEESE; *n*=10) or milk protein concentrate (MILK; *n*=10) during 4 h of recovery from a single bout of unilateral exercise in healthy, young males. t=0 min represents the time of protein ingestion. Values represent means±SD. Data were analyzed using repeated measures (Time x Group) ANOVA and separate analysis were performed when a significant interaction was detected. Bonferroni post hoc testing was used to detect differences between timepoints. *, MILK significantly different from CHEESE.

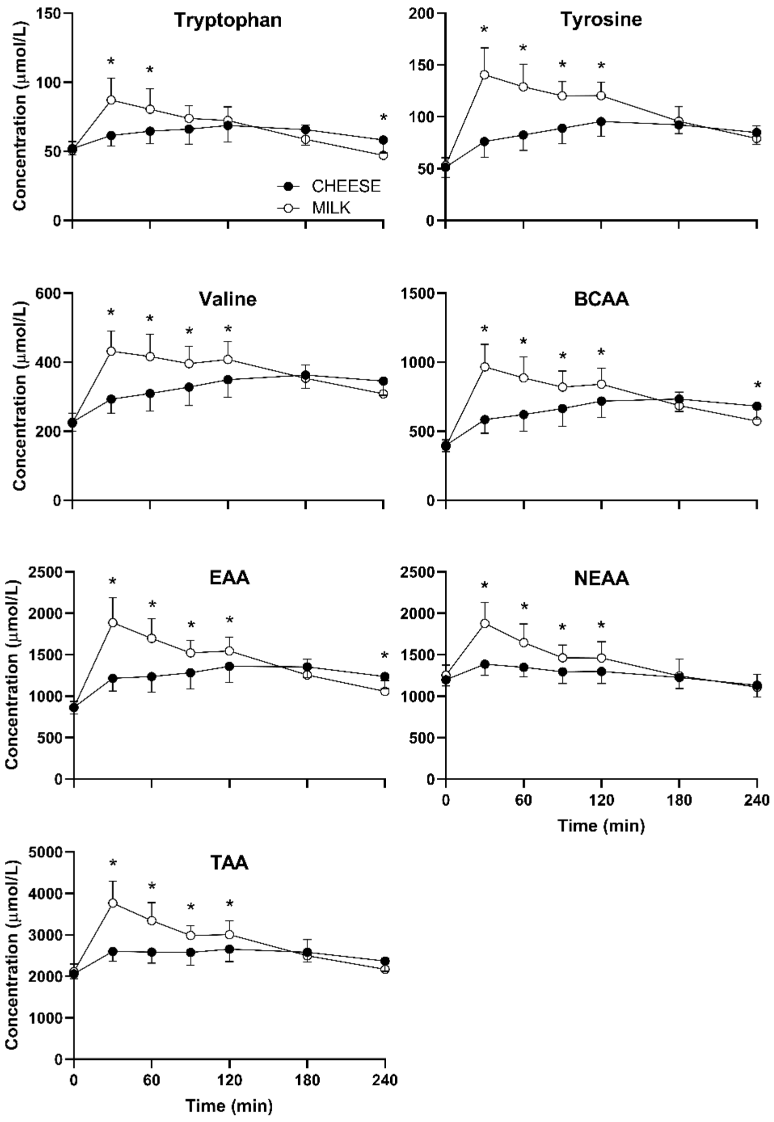

Supplemental figure 4 Plasma amino acid concentrations following ingestion of 30 g protein provided as cheese (CHEESE; *n*=10) or milk protein concentrate (MILK; *n*=10) during 4 h of recovery from a single bout of unilateral exercise in healthy, young males. t=0 min represents the time of protein ingestion. Values represent means±SD. Data were analyzed using repeated measures (Time x Group) ANOVA and separate analysis were performed when a significant interaction was detected. Bonferroni post hoc testing was used to detect differences between timepoints. BCAA, Sum of branched-chain amino acids; EAA, Sum of essential amino acids;

NEAA, Sum of non-essential amino acids; TAA, Sum of all amino acids. *, MILK significantly different from CHEESE.

REFERENCES

1. Hermans WJH, Senden JM, Churchward-Venne TA, Paulussen KJM, Fuchs CJ, Smeets JSJ, et al. Insects are a viable protein source for human consumption: from insect protein digestion to postprandial muscle protein synthesis in vivo in humans: a double-blind randomized trial. Am J Clin Nutr. 2021.
